# Optical Fiber Interferometers Based on Arc-Induced Long Period Gratings at INESC TEC

**DOI:** 10.3390/s21217400

**Published:** 2021-11-07

**Authors:** Paulo Caldas, Gaspar Rego

**Affiliations:** 1 proMetheus, Instituto Politécnico de Viana do Castelo, Rua Escola Industrial e Comercial Nun’Álvares, 4900-347 Viana do Castelo, Portugal; gaspar@estg.ipvc.pt; 2 Center for Applied Photonics, INESC TEC, Rua Dr. Roberto Frias, 4200-465 Porto, Portugal

**Keywords:** long period grating, optical fiber interferometric sensors, low-coherence interferometric, true pseudo-heterodyne technique

## Abstract

In this work, we review the most important achievements of an INESC TEC long-period-grating-based fiber optic Michelson and Mach–Zehnder configuration modal interferometer with coherence addressing and heterodyne interrogation as a sensing structure for measuring environmental refractive index and temperature. The theory for Long Period Grating (LPG) interferometers and coherence addressing and heterodyne interrogation is presented. To increase the sensitivity to external refractive index and temperature, several LPG interferometers parameters are studied, including order of cladding mode, a reduction of the fiber diameter, different type of fiber, cavity length and the antisymmetric nature of cladding modes.

## 1. Introduction

The use of optical fibers as intrinsic sensing elements for different parameters is very promising due to their set of favorable characteristics, most notably the possibility of remote and multiplexed operation [1,2,3,4,5,6,7]. Within a broad range of optical modulation mechanisms, the evanescent interaction of a guided wave with the surrounding media has great potential for environmental sensing [6,8,9,10,11,12,13]. In this context, the development of configurations with enhanced evanescent interaction capability is a necessary step towards sensor implementation in this domain. Strategies that have been explored with such purpose include side-polishing, chemical etching or tapering [6,11,14,15]. While these methods allow for greatly increased evanescent fields, their application is often associated with complex procedures and introduces fragility to the fiber sensing probe. These problems can be reduced if the change in the evanescent field is measured using interferometric schemes [16,17,18,19,20,21,22,23].

Optical interferometry is well established and associated with high precision measurements. An optical interferometer is an optical instrument in which two or more optical path lengths may be compared and the resultant intensity varies with the relative path difference, with a period equal to the optical wavelength. Thus, optical path lengths can be measured on the scale of the wavelength of light. The advent of single-mode optical fiber and related components made it possible to construct interferometers in fiber, which is equivalent to bulk-optic interferometers that are sufficiently robust to be used in practical applications outside the laboratory.

Optical fiber interferometric sensors are now the basis of a wide range of new types of measuring instruments [11,16]. In general, interferometric sensors can provide a very high level of sensitivity when the technique is used in a way that is appropriate to the measurement, although cross-sensitivity problems often arise when the devices cannot be fully isolated. Therefore, focus has been placed on R&D of optical fiber interferometric configurations that are able to maximize the sensitivity of light to variations of the medium refractive index and minimize the intrinsic sensitivity to other parameters, mostly temperature. Within this context, modal interferometers are very attractive, particularly ones with a reference path along the optical fiber core and a sensing path associated with a specific cladding mode excited by a Long Period Grating (LPG) [17,18,20]. These interferometric devices are appealing to environmental sensing, not only because they make it possible to tune the device sensitivity to refractive index variations of the fiber surrounding medium by selecting the order of the cladding mode to be excited, but also due to the reduced thermal sensitivity of the interferometer (the thermo-optic coefficients of the core and cladding modes are not substantially different).

The most common structure of this type is based on the Mach–Zehnder configuration: a twin LPG is impressed down the fiber to induce interference between the core and the selected cladding mode [24,25]. It is also possible to have an LPG-based modal interferometer in a Michelson configuration if the light is forced to cross a single LPG twice by mirroring the fiber end face after the grating. This structure, which was proposed and studied by Swart et al. [26,27], is attractive in view of its simplicity (operation in reflection) and increased interaction length, but also because it shows a better adequacy for sensor multiplexing in several situations.

In the schemes reported in the literature, the system response was recorded by monitoring the resulting fringe patterns with a spectrometer or optical spectrum analyzer (OSA) [24,26,28,29,30,31]. However, the detection of phase variation caused by the measurand in the interferometric system is a more attractive system. The detection of phase variation has the advantage of being immune to optical power drifts and provides improved sensitivity. The phase detection can be implemented with relatively low-cost optoelectronics, using coherence addressing and optical demodulation [17,20,23].

In the manuscript, the sensing properties of modal interferometers with Michelson and Mach–Zehnder topologies, associated in several cases to LPGs fabricated by the electric arc technique and phase demodulation, using an interferometric heterodyne interrogation approach for optical signal processing, was explored. It should be stressed that the choice of the LPG’s fabrication technique resides in the fact that it is a well stablished technique at INESC TEC, being low cost and versatile, enabling the fabrication of LPGs in virtually all kinds of fiber, as well as other devices, such as tapers [32]. On the other hand, arc-induced gratings can be fabricated in order to intrinsically increase their sensitivity to physical parameters such as strain, bending and external refractive index by changing the depth of the microdeformation and also due to the fact that coupling occurs to asymmetric cladding modes, which enhances the interaction of the mode field with the surrounding medium when the grating is bent [33,34].

## 2. Interferometers in Optical Fibers

Optical interferometry is associated with high accuracy measurements. An optical interferometer is an instrument in which the light physically follows two or more distinct optical paths and converts a phase change, resulting from an optical path difference to an intensity change in the detector. One of those optical paths is used as the sensing arm and the other is used as the reference arm. The easier process to implement an interferometer is to split the amplitude of the light from an optical source into two waves that travel different paths before being recombined. These interferometers are known as two-wave interferometers and the most common configurations are the Michelson and Mach–Zehnder layouts.

**Figure 1:**
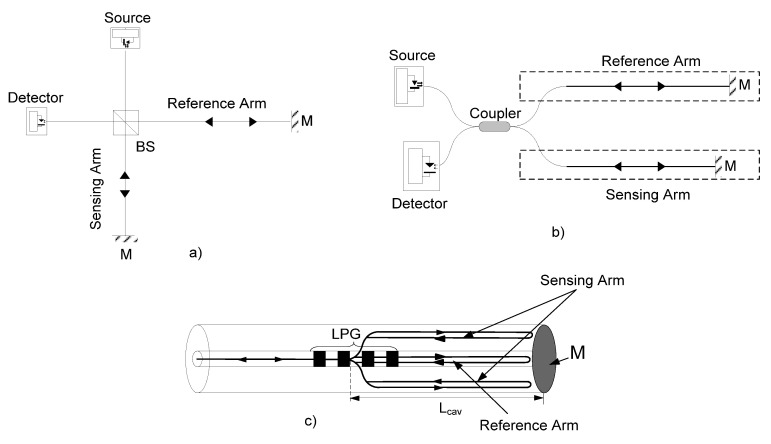
Michelson interferometer: (**a**) bulk; (**b**) optical fiber interferometer and (**c**) in-fiber LPG interferometer.

A common form of optical interferometer is the Michelson configuration, as shown in Figure 1 [26,35,36]. The light from an optical source passes through a 50:50 splitter and the divided amplitude is guided in two arms, which can be defined as the sensing arm (signal beam) and the reference arm (reference beam). The measurand modifies the phase of the signal beam, which is modified by the measurand, whereas the reference beam enjoys a constant environment. The signal and reference beams terminate in mirrors, and they are reflected back into the same path to recombine at the same 50:50 splitter that was used to divide them. The double-pass of the signal beam effectively doubles the sensitivity of the interferometer.

Figure 1 represents the three possible configurations for a Michelson interferomter: (a) represents the classical bulk configuration and consists of a beam splitter and two mirrors placed at right angles relative to each other, both of which are positioned at forty-five degrees to the beam splitter; (b) represents the configuration totally in fiber, the main difference to the bulk interferometer being that the light waves along both optical paths are confined to travel in a single-mode fiber (the fiber optic Michelson interferometer uses a directional coupler (DC) as a beam splitter, and the mirrors on the tips of each fiber provide the necessary reflection); and (c) represents the in-fiber interferometer. In this case, the beam splitter is replaced by an LPG with 3 dB losses.

To analyze the output of an interferometer, a two-wave description will be used. Consider first the Michelson interferometer shown in Figure 1. The light of the optical source splits at the beam splitter into two beams; one goes through the reference arm and the other through the sensing arm. These two beams can be described by [35]:

(1) $$\mathrm{Reference}=A_{r}e^{i\left(\omega_{L}t+2kx_{r}\right)}$$

(2) $$\mathrm{Sensing}=A_{s}e^{i\left(\omega_{L}t+2kx_{s}\right)}$$

where *A_r_* and *A_s_* are the amplitudes of the reference and the signal, *x_r_* and *x_s_* are the lengths of the reference and the sensing arms and $\omega_{L}$ is the angular frequency of the light. The phase variation in the interferometer corresponds to changes in the physical path or to changes in the index of refraction and the difference in phase between the two arms can be translated as [35]:

(3) $$\phi (t)=\phi_{r}-\phi_{s}=\phi_{o}+\Delta \phi$$

where $\phi_{r}=2kx_{r}$ and $\phi_{s}=2kx_{s}$, $\phi_{o}$ represents a quasi-static phase difference between the two waves, while the change in the relative optical paths of the two waves is reflected into Δϕ. After the recombination of the two waves on the beam splitter, the amplitude of the recombined wave is given by [35]:

(4) $$A=A_{r}e^{i\omega_{L}t}+A_{s}e^{i\left(\omega_{L}t+\phi \left(t\right)\right)}$$

The output of the interferometer is read in a detector that is sensitive to the intensity of light. The intensity and amplitude are related by:

(5) $$I\propto {\left|A\right|}^{2}=A\cdot A^{*}$$

In this case, the interferometer intensity is given by [35,36]:

(6) $$I=A_{r}^{2}+A_{s}^{2}+2A_{r}A_{s}\cos\left[\phi \left(t\right)\right]$$

or

(7) $$I=I_{r}+I_{s}+2\sqrt{I_{r}I_{s}}\cos\left[\phi \left(t\right)\right]$$

where ϕ(t) is the phase difference between the two arms and is given by:

(8) $$\phi \left(t\right)=\frac{4\pi nL_{cav}}{\lambda }$$

**Figure 2:**
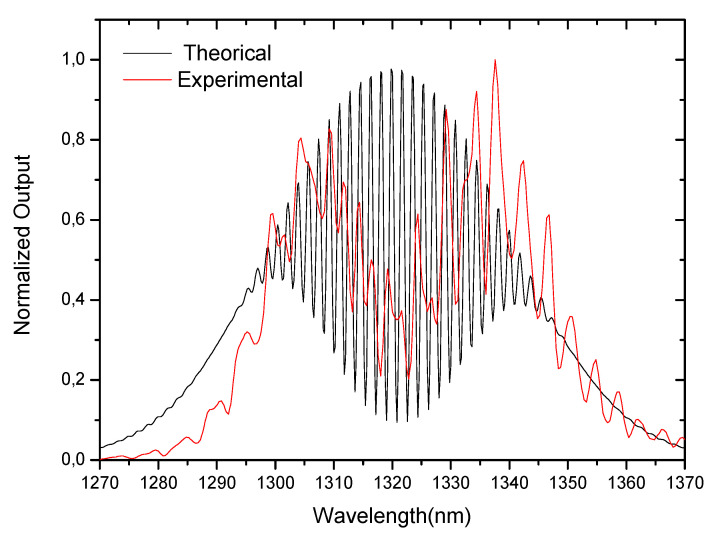
Spectrum of an LPG-assisted modal interferometer (theoretical and experimental results) for a cavity length of ~140 mm (it is involved the 4th order cladding mode).

In the LPG-based fiber modal Michelson interferometer, the LPG, with a peak depth of approximately 3 dB corresponding to the half-power point [37], couples a fraction of light to a specific cladding mode, whereas the remaining light keeps propagating in the fiber core. In this configuration, the light that propagates in the core is the reference signal and the light in the cladding is the sensing signal. At the fiber end a silver thin film reflects the light back to the LPG, which induces again cross-coupling between the core and cladding modes. In particular, the light that returns down the lead fiber has contributions from the light that propagated in the core and in the cladding modes at the sensing region, which accumulates a differential optical path delay (OPD), $\Delta nL_{cav}$, which is dependent on the measurand action. The output of the interferometer is given by Equation (7), but now the transmission through the LPG must be taken into account. The factor of transmission through the LPG for the core and cladding are given by [38,39]:

(9) $$T_{core}=\cos^{2}\left(\gamma L\right)+\frac{\delta^{2}}{\gamma^{2}}\sin^{2}\left(\gamma L\right)$$

(10) $$T_{clad}=\frac{\kappa^{2}\sin^{2}\left(\gamma L\right)}{\gamma^{2}}$$

where *L* is the length of the LPG, *k* is the coupling coefficient constant, $\gamma =\sqrt{\kappa^{2}+\delta^{2}}$ and *δ* is the detuning parameter defined by the equation

(11) $$\delta =\frac{1}{2}\left(\beta_{co}-\beta_{cl}^{m}\right)-\frac{\pi }{\Lambda }=\pi \Delta n_{eff}\left(\frac{1}{\lambda }-\frac{1}{\lambda_{D}}\right)$$

where $\lambda_{D}=\Delta n_{eff}\Lambda$ is the design wavelength for an infinitesimally weak grating. Thus, the terms *Ir* and *Is* in Equation (7) are replaced by factors proportional to $T_{core}$ and $T_{clad}$. Assuming no losses in the LPG with 3 dB insertion loss, the Equation (7) can be rewritten as follows [26]:

(12) $$I=1-4T_{core}T_{clad}\sin^{2}\left(\frac{2\pi \Delta nL_{cav}}{\lambda }\right)$$

where Δn is the effective refractive index difference between the core and the cladding and *L_cav_* is the physical length of the fiber after the LPG. The resulting output spectra for *L_cav_* ~140 mm, a LPG modulation period of 439 μm and $\Delta n_{eff}=0.0034$ can be observed in Figure 2, obtained from Equation (12). In the same figure, the spectrum obtained experimentally for a Michelson interferometer with the same characteristics is shown.

Comparing the theoretical and the experimental spectra, it can be seen that the fringe contrast is poor in practice due to the losses in the cladding, imperfection in the cleaved fiber-end and poor-quality mirror. However, the fringe periodicity is very near to the theoretical one.

**Figure 3:**
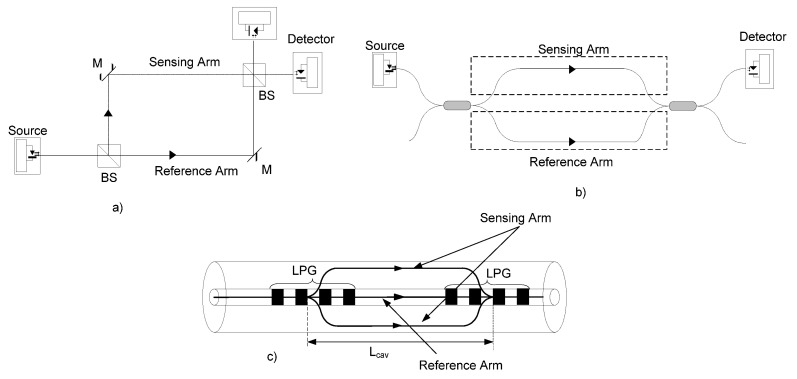
Mach–Zehnder interferometric configuration: (**a**) bulk; (**b**) optical fiber interferometer; (**c**) in-fiber LPG interferometer.

Another typical optical interferometric layout is the Mach–Zehnder configuration (Figure 3). Conceptually, the most important difference between the Mach–Zehnder and Michelson configurations is that in the Mach–Zehnder configuration, the signal and reference beams, after passing through the sensing and reference arm, are recombined by a second 50:50 splitter, where in the Michelson configuration, it is the same splitter that is used for splitting the incident optical power and also for recombining the signal and reference optical signals. In both configurations, the interference signal is detected by photodetectors, giving an electrical output proportional to the power incident upon them.

Figure 3 represents the three possible configurations for the Mach–Zehnder interferometer: (a) represents the bulk configuration and consists of two beam splitters and two reflectors; (b) represents the same configuration totally in fiber, the main difference being the fact that the light waves along both optical paths are confined to travel in a single-mode fiber (the fiber optic Mach–Zehnder interferometer uses directional couplers as beam splitters); (c) represents the in-fiber Mach–Zehnder interferometer. In this case, the two beam splitters are replaced by LPGs with 3 dB losses.

The analysis of the output of an Mach–Zehnder interferometer is similar to the Michelson interferometer, which changed only the phase difference between the two arms in Equation (7), given by

(13) $$\phi \left(t\right)=\frac{2\pi nL_{cav}}{\lambda }$$

From the analysis of Equations (8) and (13), the phase difference for the two optical configurations is expressed in slightly different ways. This difference depends on the configuration of the interferometer, verifying that the sensitivity of the Michelson interferometer is twice that of the Mach–Zehnder interferometer. This doubling of the sensitivity is due to the fact that in the case of the Michelson interferometer the light passes twice on the same path.

**Figure 4:**
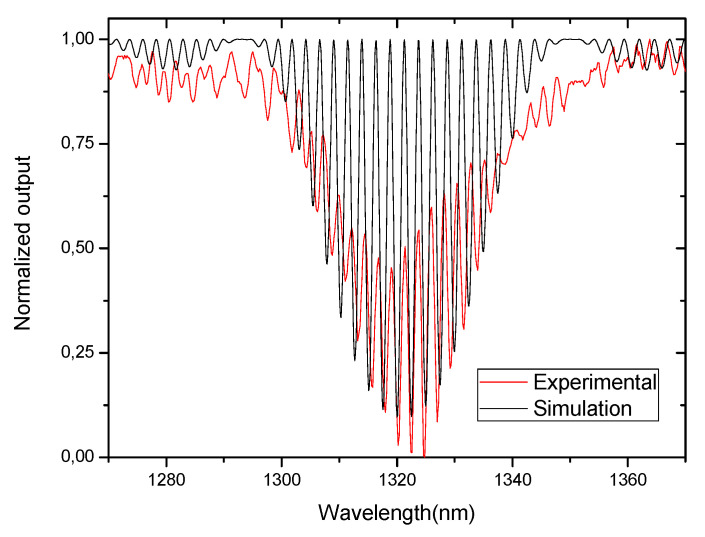
Spectrum of the LPG-based Mach–Zehnder interferometer for a cavity length of 200 mm and for LPGs operating with the 5th order cladding mode.

The LPG Mach–Zehnder interferometer is obtained by placing in series two identical LPGs, with 3-dB transmission, inscribed adjacent to each other such that an interferometer cavity of a certain length is formed between the two gratings. The first LPG couples a portion of light to cladding mode, whereas the remaining light keeps propagating in the fiber core, while the second identical LPG couple the light propagating in the cladding back to the fiber core, where interference with the light in the core mode occurs. The intensity of the Mach–Zehnder interferometer is given by Equation (7), replacing Ir and Is by values proportional to $T_{core}^{}$ and $T_{clad}^{}$, respectively, where $T_{core}^{}$ and $T_{clad}^{}$ are defined by Equations (9) and (10), respectively. Assuming no other losses in the LPGs besides the 3 dB insertion loss, Equation (7) for a Mach–Zehnder can be rewritten as follows:

(14) $$I=1-4T_{core}T_{clad}\;\sin^{2}\left(\frac{\pi \Delta n_{eff}L_{cav}}{\lambda }\right)$$

where Δn is the effective refractive index difference between the core and the cladding and *L_cav_* is the physical length of cavity. Figure 4 shows the typical spectrum of an interferometer of this type obtained from Equation (14) for *L_cav_* ~200 mm, a LPG period of 395 μm and $\Delta n_{eff}=0.00379$. In Figure 4, the experimental results obtained for an interferometer with these characteristics is also shown.

Comparing the theoretical and the experimental spectra, it can be seen that the fringe contrast is poor in the practical case because of the losses in the cladding. However, the fringe periodicity is very near to the theoretical one. When compared with the Michelson interferometer, the Mach–Zehnder interferometer is easier to implement, because it consists only of two LPGs in a series, while in the Michelson, it is necessary to place a mirror at the tip of the fiber introducing more losses in the system. However, these losses are offset by increased sensitivity that is twice that of the Mach–Zehnder interferometer. This doubling of the sensitivity is due to the fact that in the case of the Michelson interferometer the light passes twice on the same path.

**Figure 5:**
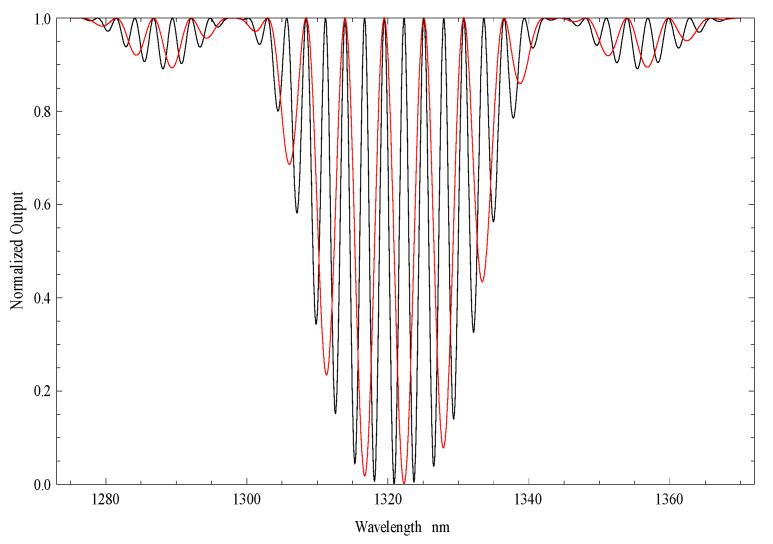
Spectrum for an LPG-based Michelson interferometer (black) and Mach–Zehnder interferometer (red) for a cavity length of 90 mm and for LPGs operating with the 5th order cladding mode.

Figure 5 shows the output intensities for a Michelson and Mach–Zehnder interferometers using Equations (12) and (14), respectively, for a *L_cav_* around 90 mm, a LPG period of 395 μm, given a 5th cladding order mode, and a $\Delta n_{eff}=0.00379$. Comparing the value of $\Delta \lambda_{Michelson}$ with $\Delta \lambda_{Mach-Zehnder}$ (the fringe periodicities) it is observed that for the Michelson interferometer is half that of the Mach–Zehnder interferometer, implying, as stated, an increased sensitivity to the Michelson structure.

## 3. Low-Coherence Interferometry

The interferometric sensors can be simply classified as a coherence interferometric sensor if the interferometer is illuminated by a laser or a monochromatic light or a low-coherence interferometric (LCI) sensor when a broadband source is used.

Low-coherence interferometry or white-light interferometry (WLI) has established itself as a powerful sensing technique in the development of a wide range of sensing systems. It was first described by Delisle and Cielo, in a paper published in 1975 [40] and demonstrated in 1976 as a possible transmission scheme to be used in optical communications [41]. This technique was first reported for use in fiberoptic sensing by Al Chalabi et al. in 1983 [42]. As with all interferometric methods, changes in the optical path are observed through interferometric fringe pattern analysis.

**Figure 6:**
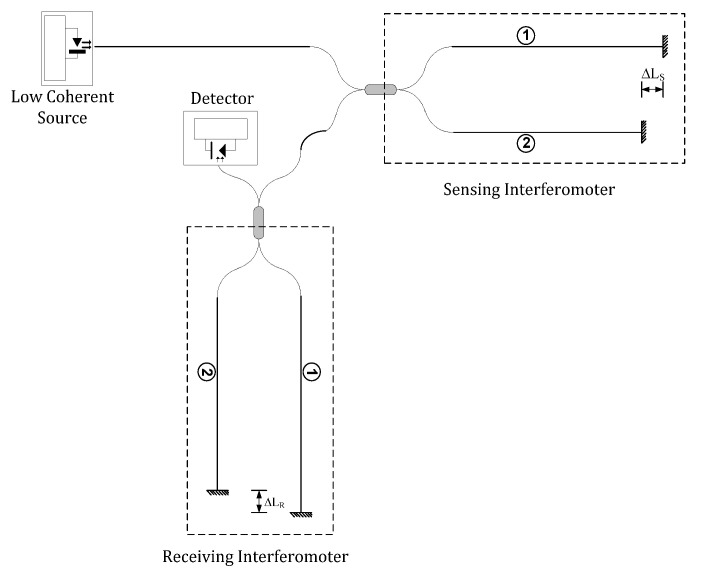
Basic low coherence interferometry system using Michelson interferometers.

LCI is an important technique for absolute remote measurement of quasi-static parameters, such as displacement, temperature, pressure, strain, and refractive index. LCI has attracted broad interest due to its stability to overcome some of the major limitations in the use of single mode laser diodes. These include a greatly reduced degree of wavelength stabilization of the source, the elimination of feedback problems in the laser cavity, the measurement accuracy is virtually insensitive to optical power fluctuations and, since the LCI system can operate with LED or SLD devices, these systems are cheap. The LCI is dependent on the relatively short coherence lengths of the source and operates by connecting the source, sensing interferometers and processing system via an optical fiber network to establish a remote sensing device. One possible configuration for a LCI is represented in Figure 6.

Basically, the LCI has a sensing interferometer and a reference or processing interferometer, and it uses a light source with a broad spectral bandwidth and a low optical coherence length, *L_c_*, much shorter than the optical path-length difference (OPD) of the interferometers, such that no interference will be observed in the time domain at the output of each individual interferometers. In Figure 6, the broadband source is launched into the optical fiber and transmitted to the sensor head interferometer (sensing interferometer). The sensing interferometer is a conventional fiber Michelson interferometer using a fiber directional coupler in which the two output ports, with the fiber ends mirrored, form the interferometer arms. Note however, that it is possible to use another interferometer configuration as a sensor (Mach-Zehnder) since the OPD is much larger than the optical coherence length of the source.

The optical path difference of the sensing interferometer, Δ*L_S_*, is larger than the coherence length of the source. The light reflected back from the mirrors is passed down the directional coupler but does not interfere. This light is connected to a second interferometer, the reference interferometer. The optical path difference, Δ*L_R_*, of this interferometer is made comparable to that of the sensing interferometer and necessarily within the coherence length of the source. Generally, in one of interferometers there is an open-air path, which is adjusted to match the optical path difference of the sensing interferometer, with the consequence that part of the radiation is brought back into temporal coherence by the interaction of the two interferometers, resulting in an interference signal that is detected on the output photodetector.

To find the output signal of this tandem interferometric arrangement, we note that the electric field at the output of the LCI is given by [43]:

(15) $$E=E_{11}+E_{12}+E_{21}+E_{22}$$

where *E_ij_* is the component of the electric field at the output arising from propagation via the *j*th arm of the sensing interferometer and the *i*th arm of the receiving interferometer, given by:

(16) $$\begin{array}{l} E_{11}=A_{11}e^{i\phi }\\ E_{12}=A_{12}e^{i(\phi +k\Delta L_{S})}\\ E_{21}=A_{21}e^{i(\phi +k\Delta L_{R})}\\ E_{22}=A_{22}e^{i[\phi +k(\Delta L_{S}+\Delta L_{R})]} \end{array}$$

where *A_ij_* is the wave amplitude of each *E_ij_*, *k* is the wavenumber, ∅ the optical phase of *E*_11_. The output optical intensity, *I*, can be obtained by taking the time averaging of Equation (15), which is the product of the overall output electric field *E* and its complex conjugate

(17) $$I=\langle E^{2}\rangle =\langle \left(E_{11}+E_{12}+E_{21}+E_{22}\right){\left(E_{11}+E_{12}+E_{21}+E_{22}\right)}^{*}\rangle$$

Assuming that the directional coupler is 50:50 and neglecting all the optical losses in the system, the output optical intensity is given by

(18) $$\begin{array}{cc} I=I_{0}\{1 & +I_{1}\left|\gamma \left(\Delta L_{S}\right)\right|\cos\left(k\Delta L_{S}\right)\\ & +I_{2}\left|\gamma \left(\Delta L_{R}\right)\right|\cos\left(k\Delta L_{R}\right)\\ & +I_{3}\left|\gamma \left(\Delta L_{S}+\Delta L_{R}\right)\right|\cos\left(k\left(\Delta L_{S}+\Delta L_{R}\right)\right)\\ & +I_{4}\left|\gamma \left(\Delta L_{S}-\Delta L_{R}\right)\right|\cos\left(k\left(\Delta L_{S}-\Delta L_{R}\right)\right)\} \end{array}$$

where *I*_0_ is the average optical power arriving at the detector, *I*_1_, *I*_2_, *I*_3_, *I*_4_ are the normalized intensities of each term in Equation (18) and |γ(∆*L*)| is the absolute value of the normalized source autocorrelation function. For a low-coherence source the autocorrelation function usually has a Gaussian profile given by [44]:

(19) $$\left|\gamma (\Delta L)\right|=e^{-\frac{\pi }{2}{\left(\frac{\Delta L}{L_{c}}\right)}^{2}}$$

Because the system is illuminated with a low coherence source, such that its coherence length is shorter than the optical path-length difference of the sensing interferometer, the interference effects will only be observed at the output of the receiving interferometer when [45]:

i. The receiving interferometer path-length difference is zero, $\Delta L_{R}=0$;
ii. The receiving interferometer path-length difference is equal to the difference in the sensing interferometer, $\Delta L_{R}=\pm \Delta L_{S}$.

**Figure 7:**
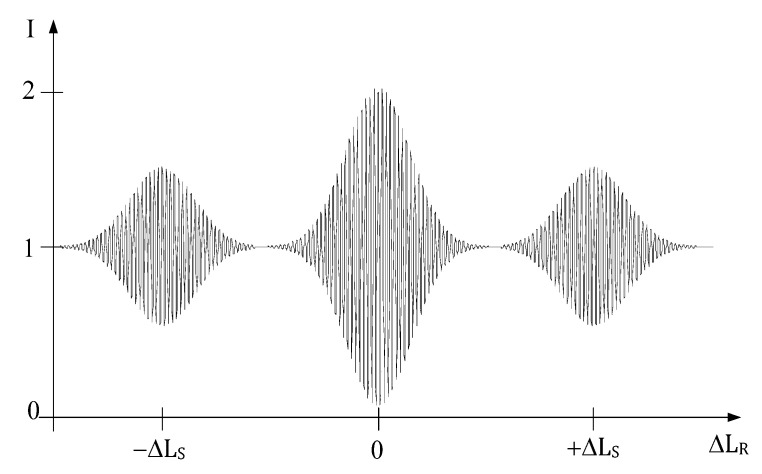
Interference fringes produced by the arrangement shown in Figure 6, as a function of $\Delta L_{R}$ in the receiving interferometer.

Figure 7 shows the output of the receiving interferometer as a function of its path-length difference when the system is illuminated by a low coherence source.

The accuracy of the measurement is governed by two factors:

(1) The ability to identify the condition corresponding to $\Delta L_{R}=\pm \Delta L_{S}$;
(2) The precision obtainable in the measurement of the optical path imbalance of the receiving interferometer.

Essentially, the LCI technique transposes the optical path difference determination from the interferometric sensor to that of the receiving interferometer. The effect of the measurand can be obtained by varying the optical path difference in the receiving interferometer, or by reading the phase of the interferometric system. As previously stated, the reading of the phase change leads to more precise measurements. To obtain this phase it is necessary to implement a dedicated phase readout technique. In the next section, a brief summary of some of these techniques will be presented.

## 4. Detection Techniques for Interferometric Fiberoptic Sensors

The basic function of the detection scheme using an interferometer as a sensor is to translate the optical phase information of an optical interferometer to an electrical signal suitable for further processing or interpretation. The detection of an optical signal is a process which is required to be accurate, stable and to have a reasonable operational range. Most detection techniques can be broadly classified as passive or active in operation [46]:

I. Passive—a scheme without feedback or one that does not require an active biasing element.
II. Active—any scheme which requires some form of feedback to the sensor or optical source, or which utilizes an electrically active optical biasing element involving tracking, scanning, or modulating mechanisms. In general, the active detection schemes require more complex systems compared to the passive ones, but the active approaches show better resolution.

In addition to this general classification, the detection and signal processing techniques for interferometric sensors can be grouped into three categories [35]:

I. Homodyne—the signal and reference waves have the same frequency when interfering with each other. In this case, the information of interest remains in the original frequency band [47,48,49,50,51];
II. Heterodyne—the optical frequency of one arm (sometimes in both) of the interferometer is shifted in order to produce a beat frequency at the output of the interferometer. Thus, the information signal appears as the phase (static or dynamic) of this beat frequency [52,53,54,55]
III. Pseudo–heterodyne—it creates a phase modulated carrier signal by modulating the wavelength of the optical source or the optical path length of one of the interferometer arms [56,57,58].

In order to conveniently analyze and measure the optical phase variations introduced in the sensing interferometer, we need a demodulation technique to have access to the optical phase, which may be one of the previously described. In this study of LPG interferometers, the Pseudo-Heterodyne Technique was chosen.

**Figure 8:**
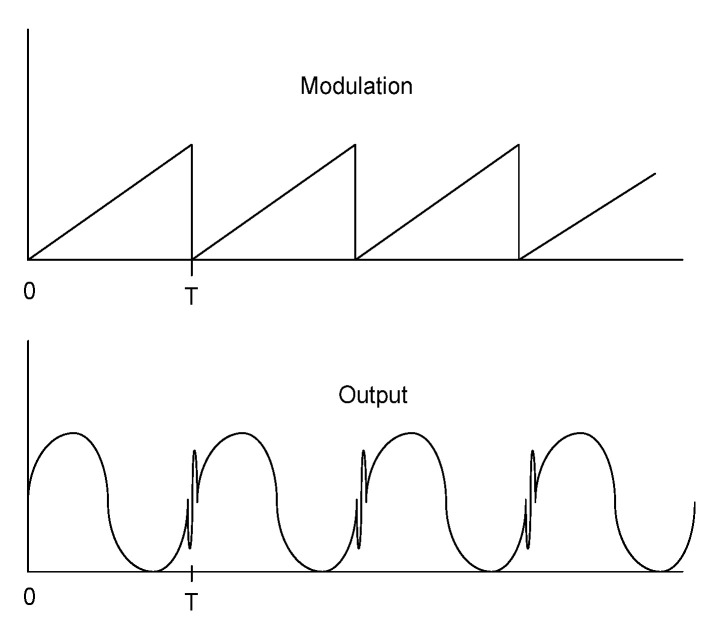
Ramp signal modulation and output when the condition $\left(\frac{d\phi }{dt}\right)T=2\pi$ is satisfied.

To implement the pseudo-heterodyne technique, a carrier signal, with a frequency *ω_c_*, is produced by modulation of the optical path of one of the arms of the receiving interferometer. To obtain the carrier signal, we use the true pseudo-heterodyne scheme. In the conventional LCI, this carrier signal is obtained by the periodic displacement of one interferometer mirror by a piezoelectric modulator or by wrapping the fiber around a ring-shaped piezoelectric (PZT) in one of the arms of the receiving interferometer, which are modulated with an electrical sawtooth (or ramp signal) waveform, generated by a function generator, that expands the PZT and the fiber, altering the optical path length (l) of the arm periodically (ramp period *T*), introducing in the interferometer a periodic modulation displacement, $\frac{dl}{dt}$. Experimentally, the amplitude of the ramp is adjusted to produce a sinusoidal waveform output signal during each ramp period, i.e., $\left(\frac{d\phi }{dt}\right)T=2\pi$. In this situation the period of the carrier signal is equal to the ramp signal (Figure 8).

In Figure 8, it is possible to see peaks in the sinusoidal wave. These peaks result from the discontinuity of the wave ramp signal that introduces a fast flyback period. This flyback can be ignored or eliminated using bandpass filtering around the ramp frequency.

The phase of this induced carrier signal is then modulated by optical path changes in the sensing interferometer in response to changes in the measurand field. The signal detected on the output photodetector is given by [46]

(20) $$I=I_{0}\{1+\left|\gamma \left(\Delta L_{S}\pm \Delta L_{R}\right)\right|\cos\left(w_{c}t+k\left(\Delta L_{S}\pm \Delta L_{R}\right)\right)\}$$

**Figure 9:**
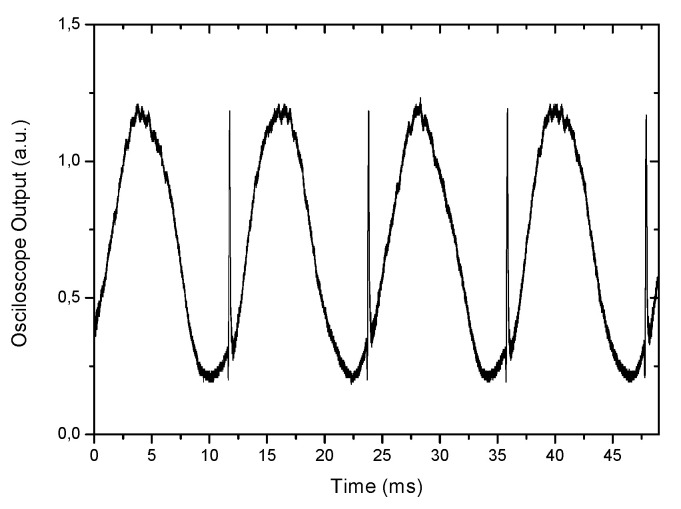
Output signal in oscilloscope for true pseudo-heterodyne.

The typical output of the detector signal given by Equation (20), when the receiver interferometer is in conditions $\Delta L_{R}=\pm \Delta L_{S}$ or $\Delta L_{R}=0$, the read in an oscilloscope is shown in Figure 9. In Figure 9, it is possible to see fast feedback due to the discontinuity of the ramp signal. The typical output signal from the lock-in amplifier when reading the phase of the pseudo-heterodyne carrier is between −180° and 180°, but the phase change is beyond those limits. Therefore, immediately after reaching a value of 180 it changes abruptly to −180. This causes an ambiguity in the output, and one needs a procedure in order to determine the point where there is the jump in the lock-in phase. Thus, after the data collection, 360 degrees has to be added every time there is a jump in phase. Then, the final phase is obtained by multiplying the number of phase jumps (*N*) by 360 degrees, plus the value of the phase before the jump, given by

(21)  Δϕ+2πN

**Figure 10:**
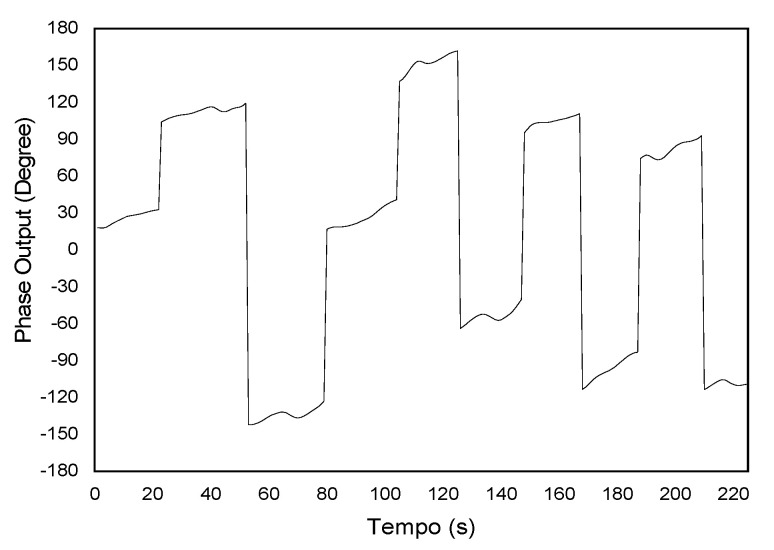
Lock-in output signal proportional to the interferometric phase variation.

**Figure 11:**
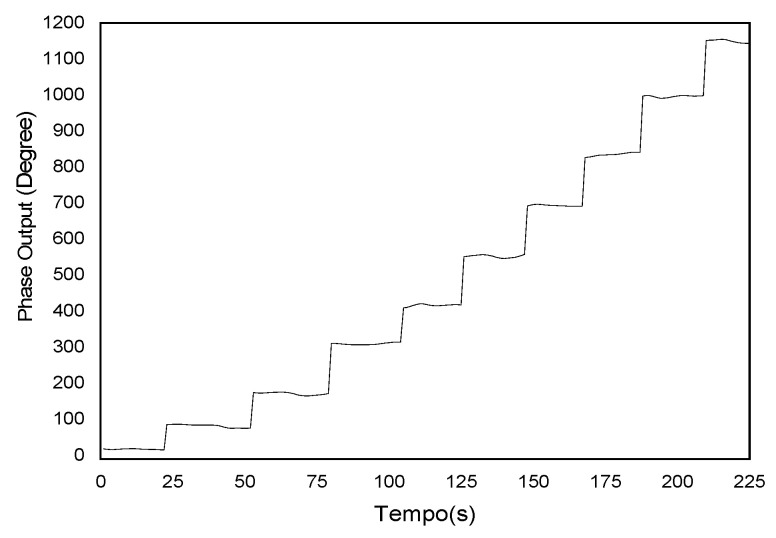
Output phase signal after removing the phase ambiguity.

Applying this treatment to the output data obtained in Figure 10 results in the value of the total phase shift (Figure 11). This data treatment will be used in all the results reported in this manuscript.

A major advantage of the LCI technique with pseudo-heterodyne processing is its relative insensitivity to the intensity and wavelength fluctuations of the optical source, in addition to its intrinsic implementation simplicity. However, the LCI technique with pseudo-heterodyne processing has some problems: one is the fact that the interference signal is very sensitive and drifts over time due to fluctuations in environmental factors such as temperature. To reduce this effect, the receiver (processing) interferometer is necessary, to create a protected environment and avoid rapid temperature changes.

In published studies, this demodulation technique has shown a large possibility to measure the optical phase variations introduced in the sensing interferometer [17,19,20,21,22,23,59,60,61,62].

## 5. LPG Interferometers Results

**Figure 12:**
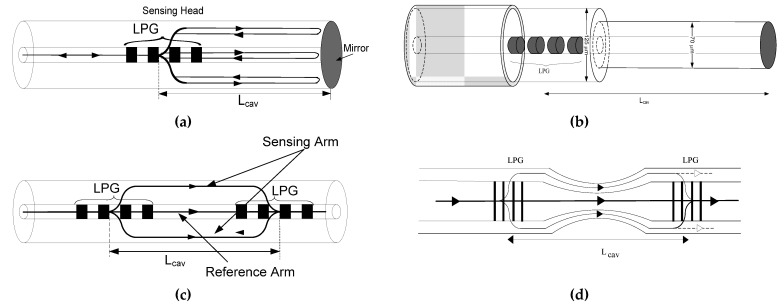
LPG interferometers: (**a**) Michelson configuration; (**b**) Michelson configuration with etching cavity; (**c**) Mach–Zehnder configuration; (**d**) Mach–Zehnder configuration with taper.

Various parameters (such as cavity length, fiber type, geometry, order of LPG modes) can influence the interferometer response. Some results will be presented in the next section where the interferometer parameters change. The experimental results obtained for the Michelson and Mach–Zehnder LPG interferometer use the experimental setup described in [17,20,23]. The light source was a super-luminescent diode (SLD), operating at 1320 nm with a FWHM spectral width of ~35 nm (coherence length *L_c_* ≈ 33 μm) to use the LCI technique. The LPG used for the Michelson and Mach Zehnder interferometers (Figure 12) was fabricated using the electric-arc technique [63] where the period of the refractive index modulation was chosen to produce a resonance wavelength at approximately 1320 nm to match the SLD central wavelength. In all experiments the contact of the LPG with the liquid was prevented to avoid shifting of the LPG resonance band. Any large shift in the LPG resonance would hinder the use of the proposed phase detection technique. In a practical system, this limitation can be easily overcome by assuring proper packaging of the LPG. Additionally, in all cases, the fiber buffer layer was previously removed.

**Figure 13:**
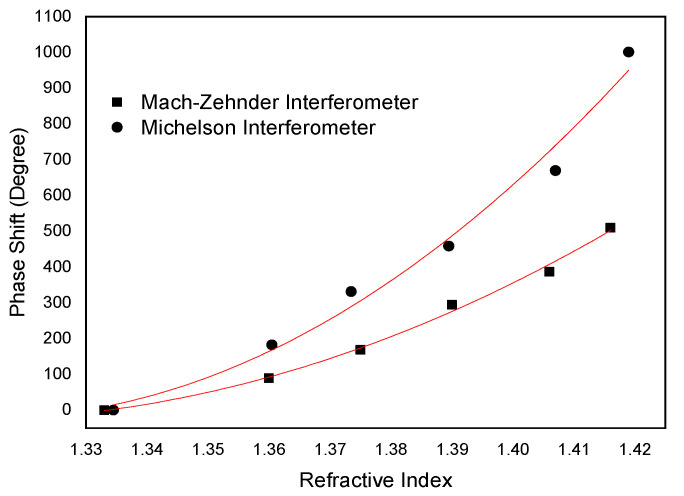
Response comparison between a Michelson and Mach–Zehnder interferometric phase changes due to refractive index variations.

The results for the Mach–Zehnder interferometer are similar to the Michelson interferometer; however, the sensitivity of the latter is twice that of the Mach–Zehnder interferometer. This doubling of the sensitivity is due to the fact that in the case of the Michelson interferometer the light passes twice on the same path. Figure 13 presents the phase response of the two interferometers, for the same *L_cav_*, to external refractive index and it can be observed that the phase shift strongly increases, in a nonlinear fashion, when the interferometer is immersed in liquids of growing density.

From the analysis of Figure 13, it can be observed that the sensitivity to external index of the Mach–Zehnder interferometer is smaller than that of the Michelson interferometer. In the refractive index region of around 1.41, values of 17919 deg/RIU for the Michelson interferometer and 8050 deg/RIU Mach–Zehnder interferometer are found, which means that for the same Lcav the sensitivity response of a Michelson interferometer is improved by a factor of 2.2 when compared with the Mach-Zehnder.

**Figure 14:**
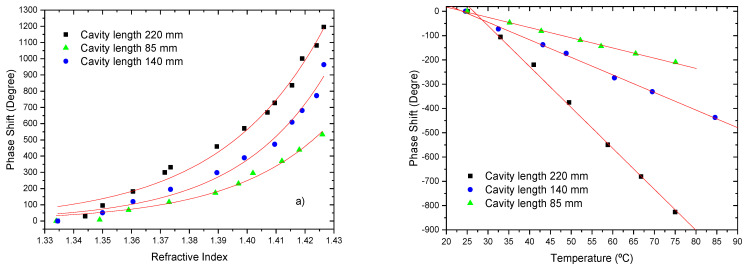
Interferometric phase changes for a 85 mm, 140 mm and 220 mm of sensing length (LPG at the 4th cladding mode): (**a**) due to refractive index variations; (**b**) due to temperature variations.

To study the effect of the cavity length, three different lengths of the cavity were used: 85 mm, 140 mm and 220 mm, measured from the middle of the LPG up to the mirrored fiber end. The 4th cladding order mode was considered for LPG operation and the Michelson configuration. Figure 14 shows the results obtained for the effect of the cavity length on the sensitivity to changes in the external refractive index and temperature.

As expected, the sensitivity to external refractive index increased with the cavity length. For instance, in the refractive index region around 1.41, values of ~$8.8\times 10^{3}$ deg/RIU, ~$2.14\times 10^{4}$ deg/RIU and of ~$2.45\times 10^{4}$ deg/RIU were obtained for the cavity lengths of 85 mm, 140 mm and 220 mm, respectively.

One of the advantages of the fiber modal interferometers is their reduced sensitivity to temperature when compared to standard fiber interferometers. The sensing head sensitivity to temperature has a negative slope, with absolute values of 4.2 deg/°C, 7.2 deg/°C and 16.8 deg/°C for the 85 mm, the 140 mm, and the 220 mm cavity length, respectively. When normalized to the fiber length this translates into a sensitivity of ~49.4 deg/°Cm, 51 deg/°Cm and 76.4 deg/°Cm for the three cavity lengths. These are reduced values, a consequence of the intrinsic differential operation of the fiber modal interferometer. For comparison, the temperature sensitivity of a standard singlemode Michelson interferometer is ~10,313 deg/°Cm, a value substantially higher [64].

**Figure 15:**
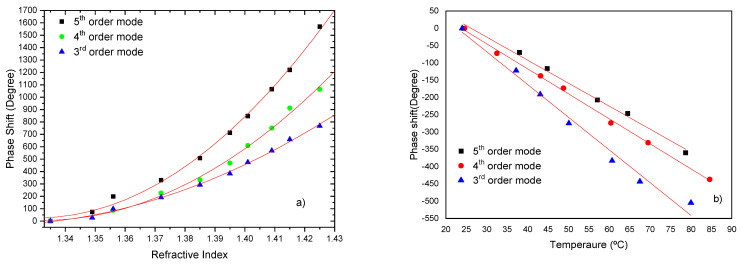
Interferometric phase changes a 140 mm sensing length and excitation of the 3rd, 4th and 5th cladding modes: (**a**) due to refractive index variations; (**b**) as function of liquid temperature.

In certain applications, the increase the length of the cavity is not possible. Instead, an increased sensitivity can be obtained by varying the order of the cladding mode associated with the LPG. This study was performed for the 3rd, the 4th, and the 5th order fiber cladding modes and the Michelson interferometer. The length of the cavity was kept constant around 140 mm. Figure 15 shows the response to changes in external refractive index (in the range of 1.33 to 1.42), in temperature and depth of the device operating with the 3rd, the 4th and the 5th order fiber cladding modes.

It can be observed that the sensitivity to external refractive index increases with the order of the cladding mode. For instance, in the refractive index region around 1.41, values of ~$1.31\times 10^{4}$ deg/RIU, ~$2.14\times 10^{4}$ deg/RIU and of ~$2.67\times 10^{4}$ deg/RIU were obtained for the 3rd, the 4th and the 5th cladding modes, respectively. The sensitivity to temperature is negative, with absolute values of 9.5 deg/°C, 7.2 deg/°C and 6.6 deg/°C for the cases under consideration. When normalized to fiber length this translates into a sensitivity of ~68 deg/°Cm, 51 deg/°Cm and 47 deg/°Cm, respectively.

It should be stressed that the electric arc technique limits the minimum modulation period that can be fabricated and, therefore, it is not possible to have access to higher order modes. Thus, an alternative approach to increase the sensitivity is to perform a reduction in the diameter of the sensing fiber by etching or by a tapper section. This reduction will encourage the interaction of the evanescent field of the cladding modes with the external ambient.

**Figure 16:**
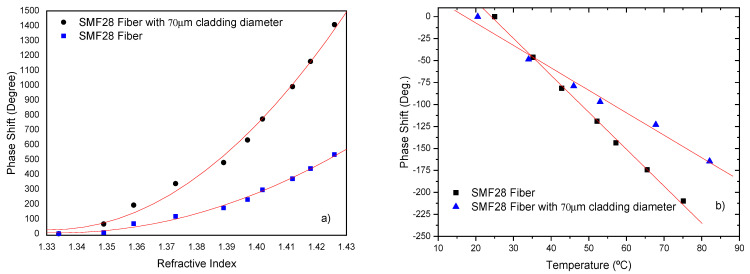
Interferometric phase changes for a 90 mm sensing length and two different sensing head fiber diameters of 125 μm and 70 μm, respectively: (**a**) to refractive index variations; (**b**) liquid temperature.

In order to characterize the effect of etching, the sensing head was tested in response to changes in the external index and the temperature. Figure 16 illustrates the enhancement on the measurement sensitivity.

From this Figure, it is observed that the sensitivity to external refractive index increases with the reduction of the fiber diameter. For instance, in the refractive index region of around 1.41, a value of ~$2.4\times 10^{4}$ deg/RIU was obtained, which means a factor of ~2.7 improvement when compared with the 125 μm standard fiber ($8.8\times 10^{3}$ deg/RIU).

The sensitivity to temperature is negative and with a magnitude of 2.5 deg/°C. When normalized to fiber length this translates into a sensitivity of ~29 deg/°Cm. This indicates another positive effect of the reduction of the fiber diameter, which is the extra reduction of the sensing head thermal sensitivity by a factor of ~ 1.7 compared to the standard SMF28 fiber (4.2 deg/°C).

**Figure 17:**
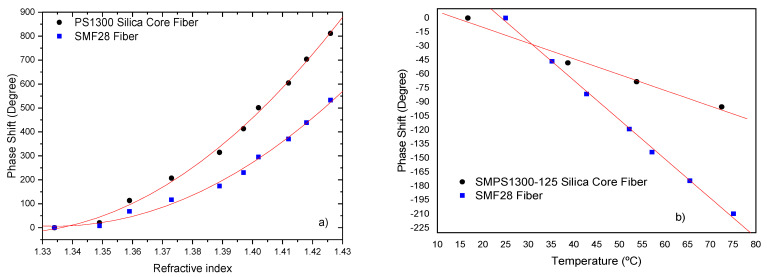
Comparison of phase changes using silica-core fiber and SMF 28 fiber (sensing length of 85 mm): (**a**) due to refractive index variations; (**b**) due to temperature.

The use of a pure-silica-core fiber is a valuable alternative to germanium doped standard fibers (SMF 28). The fiber used was the SMPS1300-125 silica-core fiber (Oxford Electronics, SMPS 1300–125 P: *D_core_* = 9 μm, *D_clad_* = 125 μm and N.A. = 0.11). Figure 17 illustrates the enhancement on the measurement sensitivity to change of external refractive index and liquid temperature which takes place when the silica-core fiber is used as the sensing head.

From Figure 17 it can be observed that the sensitivity to external refractive index increases in the pure-silica-core fiber. For instance, in the refractive index region around 1.41, a value of ~$1.24\times 10^{4}$ deg/RIU was obtained for this type of fiber. It is clear that, comparatively to the utilization of the standard SMF28 fiber ($8.8\times 10^{3}$ deg/RIU), the use of the silica-core fiber enhances the sensitivity to refractive index variations by a factor of 1.4 in the refractive index region around 1.41. For temperature variations it can be observed that the sensitivity is negative with absolute value of 1.6 deg/°C. When normalized to fiber length this translates into a sensitivity of ~18.8 deg/°C.m. Comparing this value with the one obtained for the SMF28 fiber (4.2 deg/°C) there is an extra reduction of the sensing head thermal sensitivity by a factor of ~2.6. Table 1 summarizes the results obtained for the Michelson interferometers.

Another possibility to increase the sensitivity to external refractive index is to use a taper section to enhance the evanescent field in the surround measurand region [65]. Since a tapered fiber can enhance the evanescent field, a promising possibility is to taper the fiber between the two LPGs [66,67]. The central part of the interferometer was heated by the electric arc formed between a pair of electrodes while pulling both ends of the fiber in opposite directions, either under a constant speed and tension.

**Figure 18:**
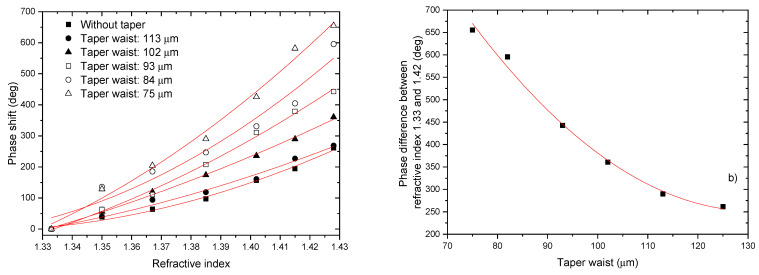
Sensing system phase changes due to refractive index variations for a tapered LPG-based Mach–Zehnder interferometer with different taper waist diameters. System phase difference versus taper waist diameter when the external medium refractive index changes from 1.33 to 1.42.

To test this possibility, four more interferometers with different taper waist diameters were fabricated. These LPG-based Mach–Zehnder interferometers were made with smaller separation between the LPGs, such that after the tapering process the final separation between the two LPGs was always ~205 mm. In this case, coupling to the 3rd order cladding mode was considered because the results obtained in the previous section show that the effect of the taper section is more significant for this internal order. The results for the system phase variations as a function of the refractive index are shown in Figure 18.

Table 2 resume the results of the Mach–Zehnder modal interferometer with different taper waist diameters.

The sensitivity increase with the reduction of the waist diameter is evident, as is better expressed by the data shown in Table 2, which gives the phase difference versus taper waist diameter when the refractive index changes from 1.33 to 1.43. Indeed, when using a taper with a waist diameter of ~75 μm, the sensitivity increases by a factor of ~1.7 when compared to the case of using a taper with a waist diameter of ~100 μm.Although the sensitivity of the Mach-Zehnder interferometer is lower than the Michelson one with the same cavity length, there are applications where it is advantageous to use Mach–Zehnder configurations. Thus, it will be useful to study processes that can increase the sensitivity of Mach–Zehnder interferometers. One possibility is to explore the antisymmetric nature of cladding modes in LPGs induced in SMF-28 optical fiber by the electric arc technique [68] to enhance and control the sensitivity to external refractive index changes in LPG-based Mach–Zehnder interferometers by a simple mechanical action on the fiber interferometer, bending the fiber of the modal interferometer.

In fact, when the fiber is bent, the effective indices of the core and cladding modes are affected differently by the photoelastic effect [69,70] and, more important in the present context, the curvature can induce a larger evanescent field of the cladding mode in the external medium, with the consequent sensitivity enhancement to changes of its refractive index.

**Figure 19:**
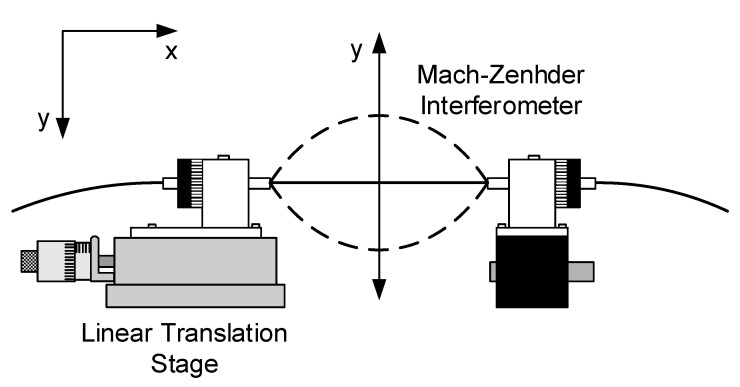
Detail of the mechanical system used to apply curvature and twist to the fiber interferometer.

**Figure 20:**
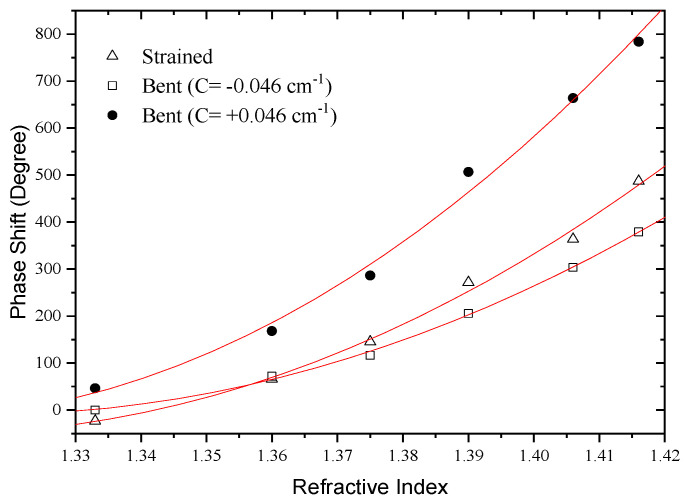
Response of the LPG-based modal interferometer for variation of the external medium refractive index when the fiber is straight and curved with values *C_o_* = ±0.046 cm^−1^.

To evaluate this possibility, the interferometer length was bent with a fixed curvature value in the *xy* plane (see Figure 19). Both positive and negative curvatures were considered (*C_o_* = ±0.046 cm^−1^). The sensitivity to external refractive index changes was then measured and compared with the situation where the fiber was kept straight. The results are shown in Figure 20.

It can be observed that the fiber bending increases the sensitivity for the case *C_o_* = +0.046 cm^−1^ and decreases when *C_o_* = −0.046 cm^−1^. In addition, it turns out that the impact of curvature on sensitivity grows steadily as the external refractive index is increased. This happens because for lower external refractive indices the index contrast between the external medium and the cladding is higher, imposing a strong confinement to the cladding modes. In the strong confinement regime, the asymmetric nature of the modes is not apparent. However, as the cladding modes are less confined the evanescent field spreads into the external medium. In such situation, any asymmetry of the cladding modes distribution is readily apparent in the device sensitivity, as demonstrated by the experimental results. It should be stressed that these results also show that the direction of curvature influences the system sensitivity to external refractive index changes, particularly for low confinement situations.

**Figure 21:**
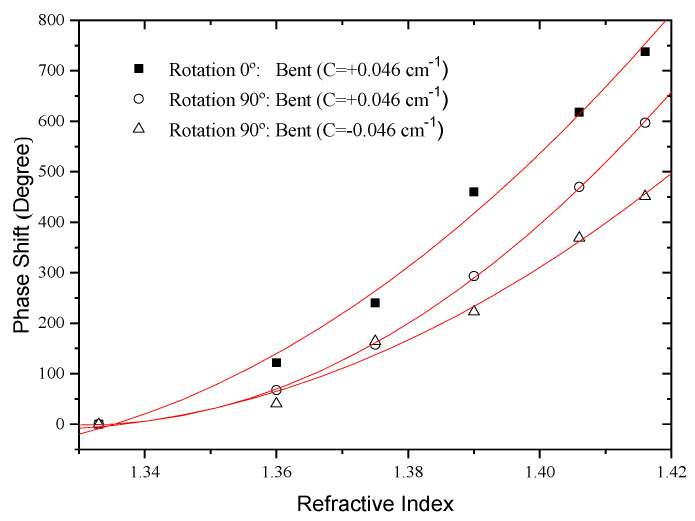
Response of the LPG-based modal interferometer for variation of the external medium refractive index when the fiber is bent in different planes (“Rotation 0°” means curvature in the *xy* plane).

The effect of bending the fiber of the interferometer in different plans on the sensing head sensitivity to external refractive index changes was also studied. For this, the fiber was rotated by 90°, through the rotation of the two graduated disks, relative to its initial position (referential from Figure 19). The first test in this position was the study of the sensitivity to external refractive index changes when the fiber was kept straight and compared with the initial position. The results are shown in Figure 21.

The results obtained for both cases were compared with those relative to curvature in the *xy* plane and *C_o_* = +0.0457 cm^−1^. Small differences are observed that may be related to the fact that the arc creates not only an asymmetric modulation of the fiber cross-section but also different annealing conditions on both sides of the fiber, which may lead to non-symmetric LP modes. Table 3 resume the results Mach-Zehnder modal interferometer with different bent planes.

## 6. Conclusions

In the present manuscript, we have reviewed the most important achievements of our research group at INESC TEC for the LPG-based interferometric sensing structures. The two configurations considered were the Michelson and the Mach–Zehnder layouts. The method used to acquire the interferometric phase shift induced from changes in the environment was described, which is based on coherence addressing and pseudo-heterodyne interrogation.

Overall, the results obtained indicate the possibility of tuning the interferometer sensitivity using different approaches, offering extra flexibility on the definition of the sensing head sensitivity to match the requirements of different applications, broadening the range of its applicability.

## Figures and Tables

**Table 1:** Resume of the results obtained with the Michelson modal interferometer.

Sensor Head	Sensitivity to	
	Refractive Index (deg/RIU)	Temperature (deg/°C)
LPG 4th order, 220 mm	2.45 × 10^4^	−16.8
LPG 4th order, 140 mm	2.14 × 10^4^	−7.2
LPG 4th order, 85 mm	8.80 × 10^3^	−4.2
LPG 3rd order, 140 mm	1.31 × 10^4^	−9.5
LPG 5th order, 140 mm	2.67 × 10^4^	−6.6
SMPS1300-125 silica fiber, 85 mm	1.24 × 10^4^	−1.6
SMF28 70 μm of diameter, 85 mm	2.40 × 10^4^	−2.5

**Table 2:** Resume of the results obtained with the Mach–Zehnder modal interferometer with different taper waist diameters.

Sensor Head	Refractive Index (deg/RIU)
MZ interfermeter Without taper	4.04 × 10^3^
MZ interfermeter Taper waist: 113 mm	4.16 × 10^3^
MZ interfermeter Taper waist: 102 mm	4.80 × 10^3^
MZ interfermeter Taper waist: 93 mm	5.07 × 10^3^
MZ interfermeter Taper waist: 84 mm	5.68 × 10^3^
MZ interfermeter Taper waist: 75 mm	8.84 × 10^3^

**Table 3:** Resume of the results obtained with the Mach–Zehnder modal interferometer for variation of the external medium refractive index when the fiber is bent in different planes.

Sensor Head	Refractive Index (deg/RIU)
MZ interfermeter strained	8.05 × 10^3^
MZ interfermeter Rotation 0°: Bent C = +0.046 cm^−1^)	1.20 × 10^4^
MZ interfermeter Rotation 0°: Bent C = −0.046 cm^−1^)	6.60 × 10^3^
MZ interfermeter Rotation 90°: Bent C = +0.046 cm^−1^)	1.16 × 10^4^
MZ interfermeter Rotation 90°: Bent C = −0.046 cm^−1^)	8.80 × 10^3^
